# Methodological Insight Into Mosquito Microbiome Studies

**DOI:** 10.3389/fcimb.2020.00086

**Published:** 2020-03-17

**Authors:** Sonia M. Rodríguez-Ruano, Eliška Juhaňáková, Jakub Vávra, Eva Nováková

**Affiliations:** ^1^Department of Parasitology, Faculty of Science, University of South Bohemia, Ceske Budejovice, Czechia; ^2^Institute of Parasitology, Biology Centre of ASCR, Ceske Budejovice, Czechia

**Keywords:** dissection, epidemiology, microbiome, mosquito, pooling, preservation, vector

## Abstract

Symbiotic bacteria affect competence for pathogen transmission in insect vectors, including mosquitoes. However, knowledge on mosquito-microbiome-pathogen interactions remains limited, largely due to methodological reasons. The current, cost-effective practice of sample pooling used in mosquito surveillance and epidemiology prevents correlation of individual traits (i.e., microbiome profile) and infection status. Moreover, many mosquito studies employ laboratory-reared colonies that do not necessarily reflect the natural microbiome composition and variation in wild populations. As a consequence, epidemiological and microbiome studies in mosquitoes are to some extent uncoupled, and the interactions among pathogens, microbiomes, and natural mosquito populations remain poorly understood. This study focuses on the effect the pooling practice poses on mosquito microbiome profiles, and tests different approaches to find an optimized low-cost methodology for extensive sampling while allowing for accurate, individual-level microbiome studies. We tested the effect of pooling by comparing wild-caught, individually processed mosquitoes with pooled samples. With individual mosquitoes, we also tested two methodological aspects that directly affect the cost and feasibility of broad-scale molecular studies: sample preservation and tissue dissection. Pooling affected both alpha- and beta-diversity measures of the microbiome, highlighting the importance of using individual samples when possible. Both RNA and DNA yields were higher when using inexpensive reagents such as NAP (nucleic acid preservation) buffer or absolute ethanol, without freezing for short-term storage. Microbiome alpha- and beta-diversity did not show overall significant differences between the tested treatments compared to the controls (freshly extracted samples or dissected guts). However, the use of standardized protocols is highly recommended to avoid methodological bias in the data.

## Introduction

The spread of vector-borne diseases, particularly those vectored by mosquitoes, is one of the main problems humanity has faced (Johnson et al., 2018). The relevance of the topic to human health has encouraged a great number of epidemiological studies in this field. However, most of the research has been focused on mosquito population distribution, early detection of invasive species, and disease agent surveillance (Kampen et al., 2015). This information is essential for keeping track of mosquito-vectored diseases, but lacks any further insight into the processes that underlie or affect the mosquito-pathogen interactions. For this reason, research focuses are shifting to the mechanisms involved in vectors' infection by pathogens and the development of new vector control strategies (Niang et al., 2018).

Microbiomes have arisen as a key factor driving many aspects of host physiology (i.e., the holobiont theory: Bordenstein and Theis, 2015; Guégan et al., 2018), including development, nutrition, survival, and competence for pathogen transmission in insect vectors (Saldaña et al., 2017). Particularly, the extent to which the microbiome can interact with arboviruses (i.e., arthropod-borne RNA viruses of the families Flaviviridae, Togaviridae, Bunyaviridae, Reoviridae, and Rhabdoviridae) has received great attention due to their epidemiological relevance (reviewed in Palmer et al., 2018). Nevertheless, most of the knowledge on microbiome-pathogen interactions in mosquitoes lacks validation from field-collected data, as it is often based on laboratory-reared mosquitoes (Minard et al., 2013; Guégan et al., 2018). In addition, large numbers of field-collected mosquitoes are processed by sample pooling to decrease research time and expenses in epidemiological studies (for a review see Engler et al., 2013).

Pooled samples allow us to track microbiome dynamics at the population level and to examine general species-specific patterns. However, pooling fails to capture the inter-individual diversity found in populations, which can be substantial and variable across vector species (Nováková et al., 2017). The resolution of inter-individual variation and individual diversity of vector-associated microbiomes has principal implications for epidemiology. The microbiome, being the more dynamic component of a holobiont, can promptly react to changing environmental conditions and drive vector adaptation and evolution. The inter-individual variation among host microbiomes is fertile ground for evolutionary novelties to arise in vector populations. The current quest to develop novel microbe-based vector control strategies thus goes through the exploration of natural microbiome variability. Understanding its role in host evolution, adaptation and physiology, including pathogen acquisition, resistance, and transmission, are key for efficient and sustainable biocontrol (Guégan et al., 2018). Although *Wolbachia*-based strategies have been successfully applied in several tropical areas as part of the World Mosquito Program (reviewed in O'Neill, 2018), consideration of the epidemiological, ecological, and evolutionary aspects of the holobiont is missing in current approaches.

The aim of this study is to demonstrate how sample pooling affects the microbiome profile obtained from wild mosquito samples, and to test different methodologies that could be applied in epidemiological studies to reduce processing costs whilst simultaneously allowing for individual specimen data collection and analysis. Particularly, we compare the microbiome alpha- and beta- diversity at different levels of pooling in two cosmopolitan species of mosquitoes, *Aedes vexans* and *Culex pipiens*. We test how different preservation methods and the use of dissected versus whole mosquitoes affects the nucleic acid yields and the diversity of the microbiome sequenced from the extracted DNA of *Aedes vexans*. Our results allow researchers in the field of vector epidemiology to choose between the proposed cost-effective methods for handling vector samples as individuals on which rigorous microbiome studies can be performed.

## Materials and Methods

### Mosquito Collection

Mosquitoes were collected in forest areas of south west part of Czech Republic (48°58′52.6″N, 14°47′56.3″E) in summer 2016 (July 20 and August 17, under similar weather conditions) using an entomological aspirator. After morphological identification, 53 female mosquitoes of *Aedes vexans* species complex were selected and divided into the different methodological categories tested (for preservation and dissection) as shown in Table 1.

**Table 1:** List of processing treatments and number of samples used in the experiment.

**Dissected tissue**	**Preservation method**	***Ae. vexans* samples**
Gut	None (Fresh)	7
Rest of body	None (Fresh)	10
Whole mosquito	None (Fresh)	11
	All Protect 4°C	6
	All Protect −20°C	5
	Ethanol 4°C	4
	Ethanol −20°C	6
	NAP 4°C	6
	NAP −20°C	5

In order to assess the pooling effect, we used already sequenced data from a previously published study in which adult female mosquitoes of 11 species were collected from 9 locations in Ontario, Canada between 2011 and 2013 (Nováková et al., 2017). Briefly, CDC ultraviolet light traps were set in urban areas from June to September each year. The collected individuals were morphologically identified to species. Individuals from each trap and date were pooled into samples containing 1–50 mosquitoes according to their taxonomy. The details of the specific data subset used are described below.

### Sample Preservation

Prior to DNA/RNA extraction (see below), the 53 mosquitoes were individually processed according to different methodologies. Whole mosquitoes were surface sterilized with absolute ethanol and then divided into different experimental groups (Table 1). The procedures used were as follows: (1) extraction the same day of capture without preservation (fresh), either (1a) processed as a whole, or (1b) dissected in sterile PBS under the stereo microscope to obtain the gut and the rest of the body separately; (2) individual preservation, without dissection, in microfuge tubes with (2a) AllProtect reagent (Qiagen), (2b) molecular grade absolute ethanol (VWR Life Sciences), or (2c) nucleic acid preservation (NAP) buffer (Camacho-Sanchez et al., 2013). Samples preserved in each reagent were stored for one week, either at 4°C or at −20°C. See Table 1 for a detailed list of the samples used in each treatment. Some samples were damaged during dissection and had to be discarded, resulting in uneven numbers for gut and rest of the body.

### DNA/RNA Extraction and Yield Assessment

After the different procedures were applied, a total of 60 samples were individually homogenized in Buffer RLT Plus (Qiagen) using sterile 1.5 mL microfuge tubes and pestles. To ensure maximum extraction yields for both DNA and RNA from the same sample, we used the gDNA Eliminator spin column from the RNA protocol (Qiagen) to separate RNA and DNA content. Subsequently, total RNA and DNA were extracted using the RNeasy Plus Micro Kit (Qiagen) and the QIAamp DNA Micro Kit (Qiagen) respectively, following manufacturer instructions. Both DNA and RNA were eluted in PCR-grade, RNase-free ultrapure water (Qiagen), and their concentrations in nanograms per microliter were measured using a NanoPhotometer (Implen GmbH), following the manufacturer's instructions for each nucleic acid.

### Microbiome Analysis

DNA from whole specimens and dissected guts was amplified according to the EMP protocol (http://www.earthmicrobiome.org/protocols-and-standards/16s/). The 16S rRNA gene amplicons were purified using AMPure XP magnetic beads (Beckman Coulter), and pooled equimolarly based on concentrations quantified using a Synergy H1 microplate reader (Biotek). One negative control for the extraction and three blanks for the reagents were included in the library preparation, as recommended by Knight et al. (2018). The raw, demultiplexed sequences produced in this work (Illumina MiSeq run, 300 cycles with v2 chemistry) are available under the ENA (European Nucleotide Archive) project number PRJEB35477.

Paired-end reads were merged using fastq_mergepairs with fastq_minovlen set to 20 from USEARCH v7.0.1001 (Edgar, 2013). Demultiplexing and quality filtering were performed in QIIME 1.9 using split_libraries_fastq.py with phred_quality_threshold set to 19 (Caporaso et al., 2010b). The resulting high-quality sequences were aligned using the QIIME implementation of Pynast (Caporaso et al., 2010a) and trimmed to an equal length of 251 bp with USEARCH. Finally, the dataset was clustered at 100% identity and this representative set of sequences was used for de novo OTU picking with the USEARCH global alignment option set to 97% identity. Each OTU was assigned to different taxonomic levels using the BLAST+ algorithm (Camacho et al., 2009) against the release 123 of the SILVA database (Pruesse et al., 2007). Singletons, very low abundant OTUs (as recommended in Bokulich et al., 2013), and all non-bacterial, chloroplast, and mitochondrial OTUs were filtered out using QIIME 1.9.

One of the blanks showed a high number of reads from a *Staphylococcus* OTU, which was absent in the other blanks and could be a specific contamination of this particular control. The second blank and the extraction negative control showed 4 OTUs and 9 OTUs, respectively. None of these OTUs represented more than 2% of the sequences in the analyzed mosquito samples. All the OTUs detected in these controls were considered contaminants, and were thus filtered out for our analyses. We also found *Spiroplasma* in the third blank, suggesting a cross-sample contamination. In fact, insect pathogens (i.e., Entomoplasmatales and Spirochaetales) were present in 28% of the samples (*N* = 50), showing high abundance (above 10%) in six of them. Since the presence of these groups may distort the profile of the affected individuals and thus any further comparisons, these OTUs were also removed from our analyses.

After this filtering step, we filtered the OTU table at a minimum of 1250 sequences per sample and rarefied at 1,000 sequences per sample to allow for resampling, even in the samples with the fewest reads, while normalizing the dataset (as recommended in Weiss et al., 2017). However, many samples from different treatments did not pass this threshold. We therefore lowered the filtering and rarefaction to 200 and 150 sequences per sample, respectively, to retain the maximum number of samples in each experimental treatment. A comparison of the microbiome profile at both rarefaction levels is shown in Supplementary Figure 1. Since all samples showed a very similar profile at both rarefaction levels, the analyses of the data set rarefied at the lower level (40 samples) are presented in the main text. When possible, the equivalent analyses for the rarefaction at 1,000 sequences per sample (23 samples) are available in Supplementary Material.

The evaluation of the pooling effect was performed on 16S rRNA data from *A. vexans* (*N* = 59) and *C. pipiens* (*N* = 67) collected in Toronto, Canada in 2012. The data used here are a subset of a previously published data set (Nováková et al., 2017) available at the European Bioinformatics Institute database (accession number ERP021438) and at https://qiita.ucsd.edu/ (ID 10815). Briefly, following the EMP protocol, the sequences were obtained in a HiSeq 2000 Illumina run (2 × 125 bp), demultiplexed using QIIME1.9, stitched and quality filtered using USEARCH software, and clustered to obtain the OTU table as described above. Taxonomic assignments for this study were performed using BLAST against the SILVA 123 database, and OTU table filtering followed the same steps as described above, with a rarefaction depth of 5,000 sequences per sample in this case. According to our previous results (Nováková et al., 2017) no contaminants needed to be filtered out from the data set. In addition to the individually processed mosquitoes (*Aedes N* = 32; *Culex N* = 12), the pooling levels used were: pools of 10 mosquitoes (*Aedes N* = 7; *Culex N* = 12), pools including between 20 and 25 mosquitoes (*Aedes N* = 12; *Culex N* = 23), and pools of 50 mosquitoes (*Aedes N* = 8; *Culex N* = 20).

Alpha-diversity indexes were calculated using QIIME 1.9. The richness of the samples (i.e., number of OTUs present) was estimated by Chao1 index, and OTU abundance and evenness in the samples was measured by Shannon index. Beta diversity analyses were performed in R (R Development Core Team, 2014) with the “biomformat” package (McMurdie and Paulson, 2019). The distance matrices were calculated using Bray-Curtis dissimilarities with the vegdist function of the “vegan” package (Oksanen et al., 2013). Core microbiomes at 100% were computed in QIIME 1.9, and Venn and Euler charts obtained using the online tool available at http://bioinformatics.psb.ugent.be/webtools/Venn/ and the upset function of “UpSetR” package (Conway et al., 2017), respectively. Additionally, these analyses were repeated using 10 random sub-samplings of our data set (*n* = 10 for each group), to check for sample size effects in our results (see Supplementary Material).

### Statistical Analyses

All statistical tests were performed in R (R Development Core Team, 2014), including outlier detection and removal (see a report of number of outliers for each analysis in Supplementary Table 1). We used the Kruskal-Wallis rank sum test (R “stats” package) to compare the yields (i.e., concentration values) and the alpha-diversity indexes among treatments. We used the adonis function in the “vegan” package (Oksanen et al., 2013) to compare the beta-diversity of the samples according to the different treatments, and performed NMDS analyses with the metaMDS function of the same package. Plots were obtained using the “ggplot2” package (Wickham, 2009).

## Results

### DNA and RNA Yields

After removing the detected outliers, the only significant differences among treatments were found according to the preservation method used when considering RNA yields (*X*^2^ = 18.93, *p* = 0.004). RNA yields did not significantly differ regardless of the body part used, including the rest of the body after dissection (*X*^2^ = 2.36, *p* = 0.307). The preservation method did not significantly affect DNA yields (*X*^2^ = 7.85, *p* = 0.249). DNA concentration differences were significant at 95% but not at 99% confidence interval when evaluating the body part used (*X*^2^ = 7.81, *p* = 0.020). See Supplementary Table 1 for a complete overview of the data and the results of the statistical tests.

### Alpha-Diversity

**Figure 1:**
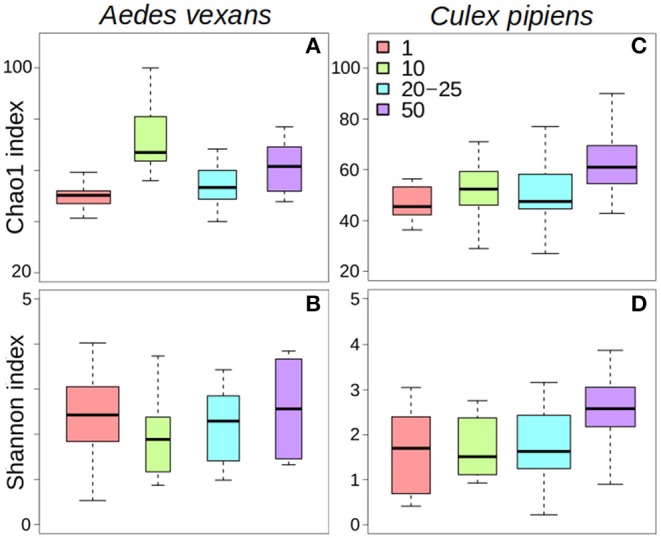
Box-plots showing the alpha-diversity of *Aedes vexans* and *Culex pipiens* microbiomes according to the number of mosquitoes pooled per sample: **(A)** Chao1 index and **(B)** Shannon index for *Aedes vexans*; **(C)** Chao1 index, and **(D)** Shannon index for *Culex pipiens*.

The comparison of the different levels of pooling revealed significant differences both for *A. vexans* and *C. pipiens*. In the first case, the richness of the samples measured by Chao1 index was significantly different among pooling levels after removing outliers (*X*^2^ = 18.935, *p* < 0.001; Figure 1A), but not Shannon index, which accounts for both OTU abundance and evenness in the samples (*X*^2^ = 2.326, *p* = 0.508; Figure 1B). In the second case, both Chao1 (*X*^2^ = 14.997, *p* = 0.002; Figure 1C) and Shannon (*X*^2^ = 10.769, *p* = 0.013; Figure 1D) indices were significantly different, at 99 and 95% confidence interval respectively, regarding the pooling levels compared. See Supplementary Table 2 for a complete overview of the data and the results of the statistical tests.

**Figure 2:**
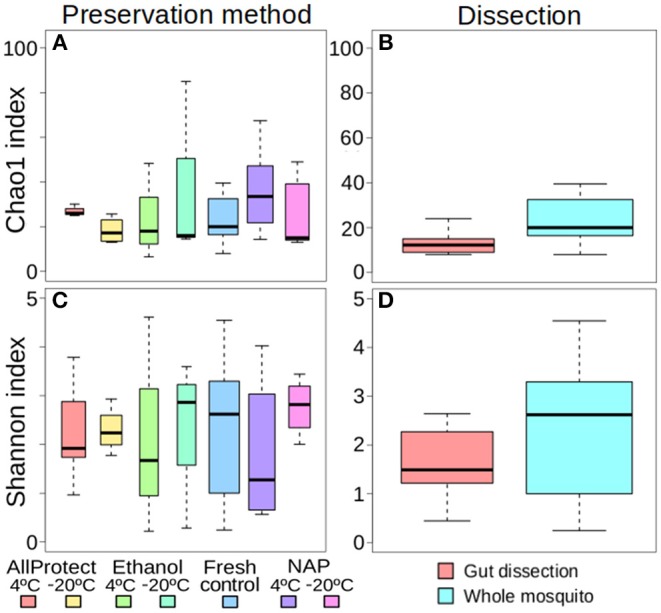
Box-plots showing the alpha-diversity of *Aedes vexans* microbiomes with the different processing treatments of this study: Chao1 index according to **(A)** preservation method and **(B)** dissection; Shannon index according to **(C)** preservation method and **(D)** dissection.

The different treatments did not affect alpha-diversity found in *A. vexans* (Supplementary Table 1). Specifically, no significant differences were found among Chao1 indices for samples preserved in different ways (*X*^2^ = 4.01, *p* = 0.676; Figure 2A), or those that originated from guts of whole specimens (*X*^2^ = 2.83, *p* = 0.093; Figure 2B; see also Supplementary Figure 2A). Shannon index did not show significant differences among treatments either regarding preservation method (*X*^2^ = 1.52, *p* = 0.958; Figure 2C) or dissection (*X*^2^ = 1.07, *p* = 0.302; Figure 2D; see also Supplementary Figure 2B). See Supplementary Table 1 for a complete overview of the data and the results of the statistical tests.

### Beta-Diversity

**Figure 3:**
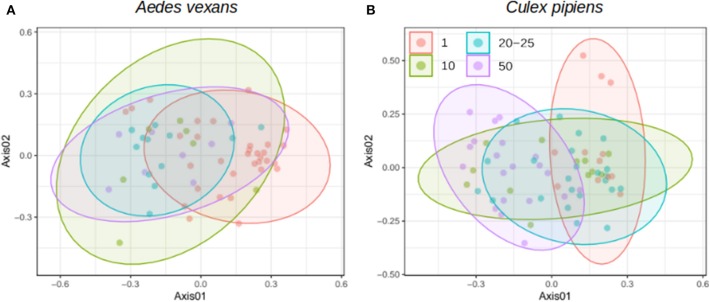
NMDS showing the beta-diversity of **(A)** *Aedes vexans* and **(B)** *Culex pipiens* microbiomes according to the number of mosquitoes pooled per sample. Confidence ellipses are shown for each group.

**Figure 4:**
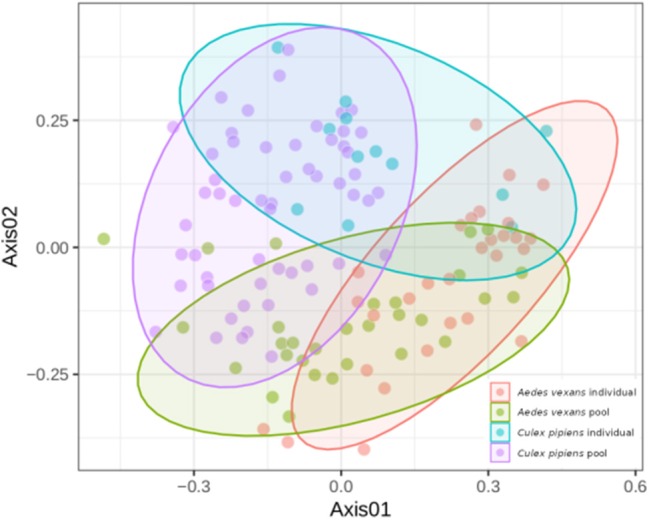
NMDS showing the beta-diversity of *Aedes vexans* and *Culex pipiens* microbiomes both from individuals and pools. Confidence ellipses are shown for each group.

When the beta-diversity was assessed for the different pooling levels compared, the differences were statistically significant for both *A. vexans* (*R*^2^ = 0.125, *p* < 0.001; Figure 3A, NMDS stress = 0.180) and *C. pipiens* (*R*^2^ = 0.131, *p* < 0.001; Figure 3B, NMDS stress = 0.200). Since individual samples were the functional control for pooling levels, additional pairwise comparisons between all groups and the control were performed. All the pooling levels differed significantly from the individual samples at 95% confidence interval, with the higher pooling levels (i.e., 20–25 and 50 individuals per pool) showing significant differences compared to the control at 99% confidence interval. The detailed results are shown in Table 2. To further explore the extent of the pooling effect on beta-diversity, we compared individual and pooled samples for both species together (Figure 4, NMDS stress = 0.218). The results showed significant differences between species (*R*^2^ = 0.121, *p* < 0.001) and between individual and pooled samples (*R*^2^ = 0.051, *p* < 0.001). The interaction of both factors was not statistically significant (*R*^2^ = 0.008, *p* = 0.302). The results for the randomly sub-sampled data sets can be found in Supplementary Figure 3.

**Table 2:** Pairwise comparisons between all pooling levels and the control (i.e., individual samples).

	***Aedes vexans***		***Culex pipiens***	
**Mosquitoes in pool vs. individuals**	***R*^**2**^**	***p***	***R*^**2**^**	***p***
10	0.06	0.0112^*^	0.08	0.0497^*^
20–25	0.1	0.0001^**^	0.07	0.0081^**^
50	0.07	0.0030^**^	0.19	0.0001^**^

Results are shown for Aedes vexans and Culex pipiens samples. Statistically significant results are indicated at 95%

(*) and 99%

(**) *confidence*.

On the other hand, no significant differences in beta-diversity were found when assessing preservation method effect (*R*^2^ = 0.214, *p* = 0.070; NMDS stress = 0.244), nor dissection (*R*^2^ = 0.103, *p* = 0.156; NMDS stress = 0.170) in *A. vexans*. When the preservation analysis was repeated, taking into account preservation buffer and storage temperature separately, temperature had a significant effect at 99% confidence interval (*R*^2^ = 0.118, *p* < 0.001), while the different buffers had none (*R*^2^ = 0.056, *p* = 0.525). The interaction of the two variables was not statistically significant (*R*^2^ = 0.040, *p* = 0.942). Since fresh samples were considered the control for preservation method, additional pairwise comparisons between all treatments and the control were performed. None of the preservation methods significantly differed from the control, and the detailed results are shown in Table 3.

**Table 3:** Pairwise comparisons of beta-diversity between all preservation treatments and the control.

**Preservation method vs. fresh samples**	***R*^**2**^**	***p***
All Protect 4°C	0.13	0.05
All Protect −20°C	0.11	0.15
Ethanol 4°C	0.08	0.64
Ethanol −20°C	0.09	0.52
NAP 4°C	0.1	0.2
NAP −20°C	0.12	0.06

*Control includes whole Aedes vexans mosquitoes extracted upon collection without any preservation step (i.e., freshly extracted)*.

### Core Microbiomes

**Figure 5:**
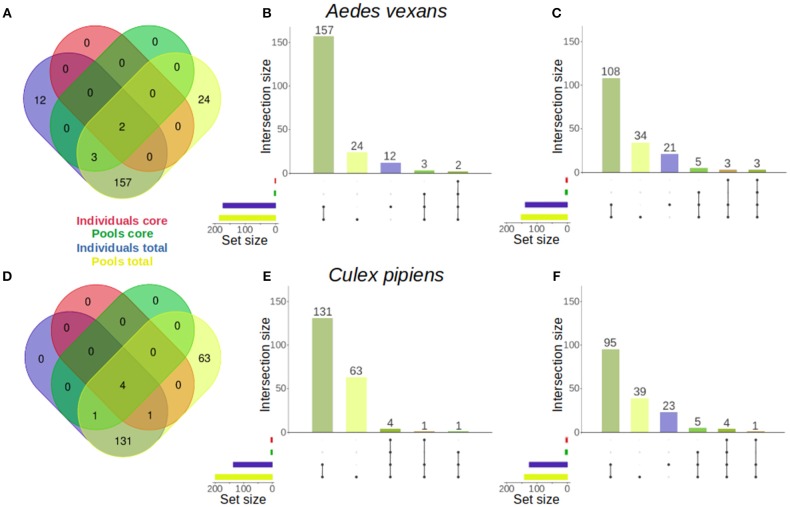
Venn diagrams comparing the number of OTUs present in individual and pooled samples, both in total and in the core microbiome of **(A)** *Aedes vexans* and **(D)** *Culex pipiens* full data sets. Proportional representations (“Euler grids”) of the same information are given for the full data set **(B,E)** and the average values of the randomly sub-sampled data sets **(C,F)** of each species.

The specific effect of pooling on the retrieval of total OTUs and core microbiome components was assessed for *A. vexans* and *C. pipiens*. The core microbiomes (i.e., OTUs present in all samples) were plotted against the total number of OTUs for both the individual samples and the pooled ones, to show the amount of unique microbiome members as well as the representation of the core microbiome retrieved by each group (Figure 5). The results for the randomly sub-sampled data sets can be found in Supplementary Figure 4.

In *A. vexans* individuals, the total number of OTUs found was 174, compared to the 186 OTUs found in pooled samples. Out of those, 12 (6.9%) and 24 (12.9%) were unique to individual and pooled samples, respectively, and 157 were common to both groups. The core microbiome of individual samples comprised two (1.1%) OTUs, *Pseudomonas* sp. and an unclassified Methylophilaceae, while the core microbiome of pooled samples comprised up to five (2.7%), including the previous two plus *Enterobacter* sp., *Serratia* sp. and a second *Pseudomonas* sp. (see Figures 5A–B; for an overview of the sub-sampled data sets average results, see Figure 5C).

In *C. pipiens* individuals, the total number of OTUs found was 137, compared to the 200 OTUs found in pooled samples. Out of those, 63 (31.5%) were unique to pooled samples, while all the 137 OTUs from individual samples were also found in the pools. The core microbiome of individual samples comprised five (3.6%) OTUs: *Wolbachia, Enterobacter* sp., two *Pseudomonas* sp., and an unclassified Methylophilaceae. The core microbiome of pooled samples included also five (2.5%) OTUs: four present in the individual samples core and another unique: *Tumebacillus* sp. (see Figures 5D–E; for an overview of the sub-sampled data sets average results, see Figure 5F).

## Discussion

Pooling is expected to bias the observed diversity of the microbiome, particularly when inter-individual variability is high in the analyzed populations. This is the case in mosquitoes, for which previous studies identified several eco-physiological factors affecting the microbial community composition (Minard et al., 2013). Microbiome data is *per se* highly dimensional (i.e., many categories), sparse (i.e., dominated by zero values) and compositional (i.e., absolute abundances are unknown) (Tsilimigras and Fodor, 2016). These complex microbiome characteristics pose a general challenge to microbiome data analysis that is only deepened with sample pooling. Each individual pooled adds on categories (e.g., infrequent taxa) and skews the relative abundances in different, unknown directions (either toward dominance of particular OTUs or evenness). This may cause, for instance, an increase of richness due to additive occurrence of rare taxa as previously discussed for tick microbiomes (Clow et al., 2018). It can also affect the beta-diversity metrics (i.e., pool inter-variability) when alpha-diversity variance increases due to differential pool intra-variability. Our results show a clear shift between the microbiome of mosquitoes processed as individuals and samples coming from pools of variable number. Along with other studies revealing microbiome differences between individual and pooled samples (e.g., in humans: Aguirre et al., 2014; and chigger mites: Chaisiri et al., 2019), this emphasizes the need for careful interpretation of results. Both the microbiome alpha- and beta- diversity of the mosquitoes used here, *Aedes vexans* and *Culex pipiens*, were significantly different between individual and pooled samples. These differences occurred independently of the species considered, and were comparable to those found between samples of the different species, suggesting the effect of pooling is considerable. We also show that the core microbiome is overestimated when using pools. For example, OTUs that may be abundant in only a few individuals could be retrieved in all pools and be included in the core microbiome of the study group. This would alter the conclusions made about potentially interesting (i.e., functionally relevant) members of the microbiome and their interactions.

Whole specimens are usually pooled in surveillance studies to screen mosquito samples for viruses using RNA and RT-PCR techniques (e.g., Čabanová et al., 2019). However, individual dissection of particular tissues (i.e., guts) is the reference methodology to study site/organ specific microbiomes (e.g., Muturi et al., 2019). This approach is, in many cases, technically complicated (i.e., when working with very small organisms) and/or logistically limiting (i.e., when large numbers of individuals have to be processed in a limited amount of time and/or with restricted resources). Since the main aim of this study was to find a feasible way to simultaneously conduct disease surveillance and obtain individual microbiomes in disease vectors, the number of individuals to dissect is the main limiting factor in this context. Our results show that, although the use of whole specimens causes an increase in the overall microbiome alpha-diversity compared to the dissected guts, the differences are not significant. The increase in diversity is most likely due to the detection of bacteria present in other parts of the body. However, the non-gut bacteria should be relatively much less abundant, and in fact, we did not find significant differences when assessing the bacterial composition of the mosquito microbiome (i.e., beta-diversity) in dissected guts and whole body samples. Such an assumption does not apply to systems in which endosymbionts associated with specific tissues like *Asaia, Rickettsia, Wolbachia, Spiroplasma*, or other reproductive manipulators are found (Duron et al., 2008; Segata et al., 2016). Nonetheless, these endosymbionts can be easily identified in the data and filtered out if necessary (e.g., *Wolbachia*: Nováková et al., 2017; Hegde et al., 2018). For insects lacking more specialized symbionts gut bacteria amplify preferentially as shown in *Drosophila* gut microbiomes, successfully described using whole specimens (Wong et al., 2013). Similarly, Whitaker et al. (2016) found no differences for two particular OTUs of interest when comparing whole specimens and dissected guts of lycaenid butterflies. Among blood-feeding vectors, comparable results were previously obtained for kissing bugs (Rodríguez-Ruano et al., 2018) and *Anopheles* mosquitoes (Coon et al., 2014), for which the microbiome profiles of dissected guts and whole bodies/abdomens did not differ significantly. Along with the results presented here, these findings collectively support the use of whole specimens in epidemiological-microbiome studies, saving the difficulties and costs of dissecting every individual prior to DNA/RNA extraction. Particular attention should be payed to the washing and surface sterilization steps prior to the extraction to avoid as much as possible external contaminants. Even though the effect of surface microbes in the whole microbiome assessment may be minimal as well (Hammer et al., 2015), the method used for surface sterilization can impact the microbiome diversity retrieved (Binetruy et al., 2019).

Methodology optimization also involves sample preservation. All methods evaluated here were able to preserve DNA (i.e., needed for microbiome profiling) and RNA (i.e., needed for virus screening), in agreement with previous studies performed with different kinds of samples (e.g., freshwater insects: Astrid et al., 2016; and mammalian blood and tissues: Camacho-Sanchez et al., 2013). Significant differences among treatments were found for RNA yields, but not for DNA. The highest extraction yields were obtained using preservation in NAP buffer at 4°C for both DNA and RNA, followed by All Protect at 4°C and absolute ethanol at 4°C, respectively. The better performance of non-freezing storage conditions may be a result of avoiding freezing-thaw cycles during sample manipulation. The finding of absolute ethanol as an efficient RNA preservative may seem surprising, but it has previously been shown for insect larvae and nymphs (Astrid et al., 2016). In general, our results agree with previous studies where NAP buffer was found as an efficient nucleic acid preservative for various samples (e.g., rat tissues: Camacho-Sanchez et al., 2018; and frog tissues Montero-Mendieta et al., 2017), including those for microbiome studies (e.g., fecal samples: Menke et al., 2017). Nevertheless, we faced difficulties fully submerging the mosquito specimens in NAP buffer. Different concentrations of glycerol (25–50%) added during preparation resulted in salt precipitation of the solution. We thus encourage future attempts on the development of efficient laboratory-made buffers that would overcome this issue and facilitate their massive usage, being an easy to prepare and more economic alternative to commercial buffers.

Almost all preservation treatments produced a slight increase in the OTU richness found in the samples compared to the control (i.e., specimens freshly extracted upon collection), with the exception of All Protect combined with freezing at −20°C. These results are contrary to those obtained by Menke et al. (2017), yet the differences found in our study are not significant. In addition, the control treatment they used involved sample freezing. Here we consider freezing as a preservation method itself, which our results show has a strong effect. Additionally, we found the OTU abundance and evenness to be lower overall with all the preservation methods when compared to the control, with the exception of NAP buffer at −20°C. These differences were non-significant in all cases, allowing us to conclude that specimen preservation method does not strongly affect the retrieved alpha-diversity of bacterial communities in the conditions tested in our study. On the other hand, preservation method did affect the beta-diversity observations. The storage temperature was more important in determining the significant differences found than the preservation buffer used. Menke et al. (2017) found a similar effect of their different preservation treatments, with a strong effect of storage temperature irrespective of the preservation buffer used. Nevertheless, none of the preservation buffers combined with storage at 4°C or freezing at −20°C significantly differed from the freshly extracted controls in our beta-diversity analyses. The differences found in the global test (i.e., when comparing all treatments) occurred among preservation treatments. This means that each method can quite accurately reflect the actual diversity of the samples, but different preservation methods can act as a confounding factor in the analyses performed. Our results highlight the importance of using the same preservation technique throughout a study, in order to avoid the bias in beta-diversity observed when different preservation methods are employed.

In summary, we confirm that sample pooling distorts the real picture of the mosquito microbiome. An accurate description of the microbiome requires, thus, the use of individual samples. Furthermore, the inter-individual variations and direct interactions between different microbiome components and transmitted pathogens, as well as their effect on host ecophysiology, can only be clarified at the individual level. We propose alternatives to optimize the cost-efficiency of the protocols to assess the microbiome of epidemiologically relevant vectors (such as mosquitoes). In general, the OTUs we observe in the mosquito microbiome are congruent with previous reports (Muturi et al., 2017; Nováková et al., 2017). Our analyses show there are no major effects on the diversity and composition of the microbiome posed by the use of whole specimens vs. dissected guts, or when combining any of the preservation buffers tested with short-term storage at 4°C or −20°C. However, the lack of a priori power analysis and the small sample size remaining after data processing combined with the high variability found in the mosquito microbiome limits the extent of our conclusions. Based on the obtained results we recommend the use of whole specimens and inexpensive preservative reagents like NAP or absolute ethanol, which do not require the samples to be stored in the freezer in the short term (i.e., during field sampling). This methodology allows for valid assessments of the bacterial microbiome alpha- and beta-diversity, while providing enough material for pathogen screening both using DNA (e.g., for nematodes and protists) and RNA (e.g., for arboviruses). In addition, it allows for the assessment of other components of the microbiome (i.e., DNA-based mycobiome and RNA-based viriome). Finally, we highlight the particular importance of using standardized methodology for sample processing and preservation when possible, in order to minimize methodological bias in microbiome studies.

## Data Availability Statement

The datasets generated for this study can be found in the European Nucleotide Archive (ENA) under project number PRJEB35477 (https://www.ebi.ac.uk/ena/browser/view/PRJEB35477).

## Author Contributions

SR-R and EN designed the study and drafted the manuscript. SR-R, EJ, and JV managed the field collections and performed the experiments. SR-R generated the data and performed the data analyses. All authors read and contributed to the final text.

### Conflict of Interest

The authors declare that the research was conducted in the absence of any commercial or financial relationships that could be construed as a potential conflict of interest.
