# Supplementary material for: Methodological Insight Into Mosquito Microbiome Studies
Source: Front Cell Infect Microbiol. 2020 Mar 17;10:86. doi: 10.3389/fcimb.2020.00086 (PMC7089923; doi:10.3389/fcimb.2020.00086)
Supplement: Supplementary file 1 [file Table_1.docx]

Supplementary Material

|  | DNA  (ng/ul) | RNA  (ng/ul) | Chao1 index  (rarefaction 150) | Chao1 index  (rarefaction 1000) | Shannon index  (rarefaction 150) | Shannon index  (rarefaction 1000) |
| --- | --- | --- | --- | --- | --- | --- |
| Whole fresh | 11 (0): 18.09 (15.68) | 10 (1): 8.64 (6.12) | 8 (0): 23.17 (10.66) |  | 8 (0): 2.33 (1.46) |  |
| All Protect 4 ºC | 6 (0): 22.67 (13.24) | 5 (1): 4.32 (1.58) | 3 (2): 27.04 (2.71) |  | 5 (0): 2.25 (1.09) |  |
| All Protect -20 ºC | 5 (0): 19.12 (6.92) | 5 (0): 2.00 (0.94) | 4 (0): 18.27 (5.93) |  | 4 (0): 2.29 (0.48) |  |
| Ethanol 4 ºC | 4 (0): 21.38 (17.49) | 4 (0): 13.40 (5.82) | 3 (0): 24.28 (21.61) |  | 3 (0): 2.17 (2.24) |  |
| Ethanol -20 ºC | 5 (1): 12.10 (3.83) | 6 (0): 10.81 (11.06) | 3 (0): 38.50 (40.28) |  | 3 (0): 2.25 (1.74) |  |
| NAP 4 ºC | 6 (0): 32.58 (14.50) | 6 (0): 15.93 (8.25) | 6 (0): 36.29 (19.71) |  | 6 (0): 1.80 (1.40) |  |
| NAP -20 ºC | 4 (1): 14.05 (15.20) | 4 (1): 6.50 (3.35) | 5 (0): 26.03 (16.85) |  | 4 (1): 2.77 (0.60) |  |
| **Test results (X^2^; p)** | **7.85; 0.2490** | **18.93; 0.0043**** | **4.01; 0.6757** |  | **1.52; 0.9584** |  |
| Whole fresh | 11 (0): 18.09 (15.68) | 10 (1): 8.64 (6.12) | 8 (0): 23.17 (10.66) | 5 (0): 23.10 (7.33) | 8 (0): 2.33 (1.46) | 5 (0): 2.00 (1.46) |
| Gut dissection | 6 (1): 7.89 (4.66) | 6 (1): 12.93 (3.72) | 6 (0): 13.42 (5.94) | 5 (0): 23.20 (8.35) | 6 (0): 1.59 (0.78) | 3 (2): 1.45 (0.24) |
| Rest of body | 10 (0): 29.81 (20.44) | 10 (0): 15.56 (13.60) |  |  |  |  |
| **Test results (X^2^; p)** | **7.81; 0.0202*** | **2.36; 0.3069** | **2.83; 0.0926** | **0.01; 0.9168** | **1.07; 0.3017** | **0.20; 0.6547** |

**Supplementary Table 1. Overview of the data and the results of the statistical tests for the comparison of the different processing methods.** For each variable measured (DNA and RNA concentration, alpha-diversity indexes Chao1 and Shannon), the table shows Number of analyzed samples per treatment (number of removed outliers): Mean (Standard Deviation) and the results of each statistical test (X^2^; p), indicating the statistically significant results for 95% (*) and 99% (**) confidence.
