# Supplementary material for: Methodological Insight Into Mosquito Microbiome Studies
Source: Front Cell Infect Microbiol. 2020 Mar 17;10:86. doi: 10.3389/fcimb.2020.00086 (PMC7089923; doi:10.3389/fcimb.2020.00086)
Supplement: Supplementary file 2 [file Table_2.docx]

Supplementary Material

|  | Mosquitoes in pool | Chao1 index | Shannon index |
| --- | --- | --- | --- |
| *Aedes vexans* | 1 | 20 (12): 49.81 (4.04) | 32 (0): 2.35 (0.89) |
|  | 10 | 7 (0): 73.19 (15.93) | 7 (0): 1.94 (1.01) |
|  | 20-25 | 10 (2): 54.49 (9.16) | 12 (0): 2.14 (0.81) |
|  | 50 | 8 (0): 61.23 (10.44) | 8 (0): 2.57 (1.08) |
| **Test results (X**^2^**; p)** |  | **18.94; 0.0003**** | **2.33; 0.5076** |
| *Culex pipiens* | 1 | 12 (0): 47.10 (6.52) | 12 (0): 1.64 (0.91) |
|  | 10 | 12 (0): 51.83 (11.70) | 12 (0): 1.68 (0.67) |
|  | 20-25 | 23 (0): 50.12 (12.06) | 23 (0): 1.75 (0.83) |
|  | 50 | 20 (0): 62.91 (13.20) | 20 (1): 2.48 (0.71) |
| **Test results (X**^2^**; p)** |  | **15.00; 0.0018**** | **10.77; 0.0130*** |

**Supplementary Table 2.** **Overview of the data and the results of the statistical tests for the comparison of the different pooling levels.** For each alpha-diversity index, Chao1 and Shannon, the table shows Number of analyzed samples per treatment (number of removed outliers): Mean (Standard Deviation) and the results of each statistical test (X^2^; p), indicating the statistically significant results for 95% (*) and 99% (**) confidence.
