# Supplementary material for: Methodological Insight Into Mosquito Microbiome Studies
Source: Front Cell Infect Microbiol. 2020 Mar 17;10:86. doi: 10.3389/fcimb.2020.00086 (PMC7089923; doi:10.3389/fcimb.2020.00086)
Supplement: Supplementary file 3 [file Image_1.pdf]

frontiers

Supplementary Material

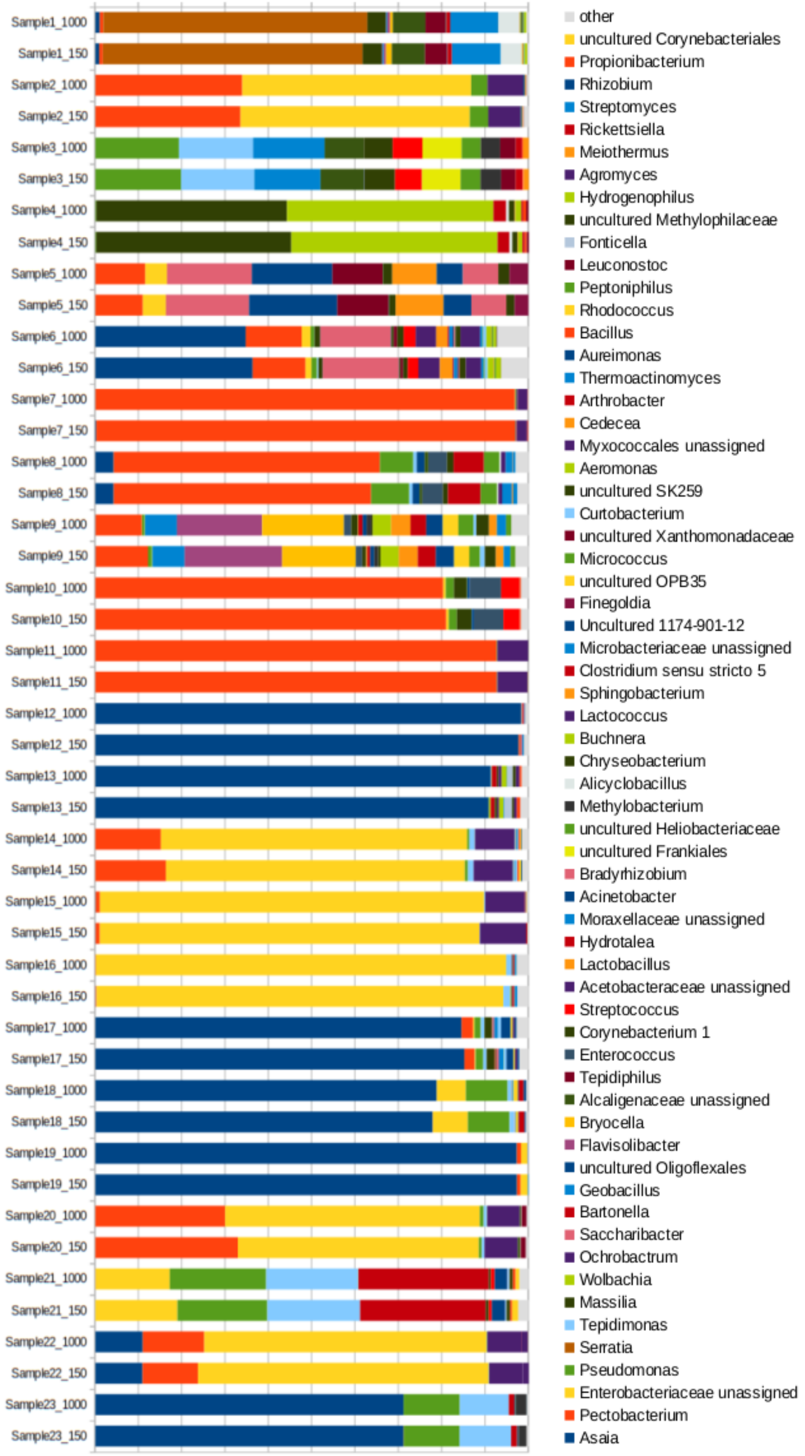

Supplementary Figure 1. Comparison of the microbiome taxonomic profiles of *Aedes vexans* samples when different rarefaction levels were used. For each sample, the OTU-level profiles based on rarefactions at 1000 and 150 sequences per sample, respectively, are shown side to side for easier comparison.
