# Supplementary material for: Methodological Insight Into Mosquito Microbiome Studies
Source: Front Cell Infect Microbiol. 2020 Mar 17;10:86. doi: 10.3389/fcimb.2020.00086 (PMC7089923; doi:10.3389/fcimb.2020.00086)
Supplement: Supplementary file 4 [file Image_2.pdf]

Supplementary Material

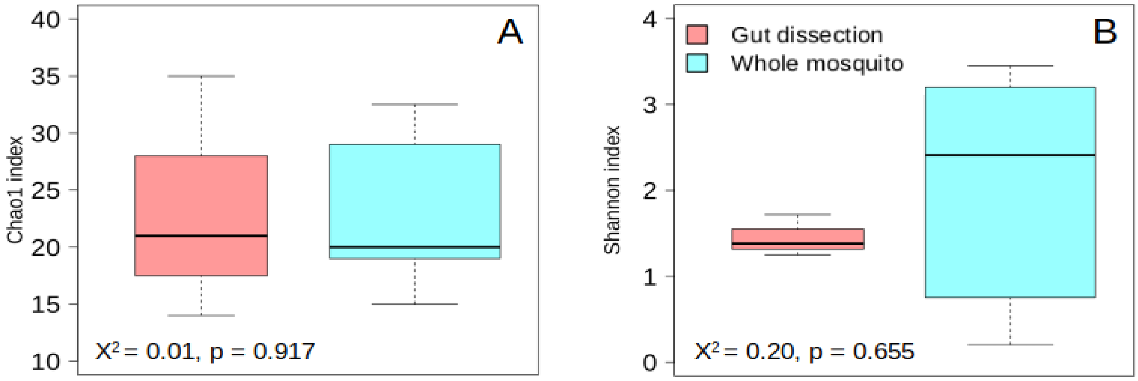

Supplementary Figure 2. Box-plots showing the alpha-diversity of *Aedes vexans* microbiomes (using 1000 sequences per sample) according to the body part used: A) Chao1 index and B) Shannon index.
