# Supplementary material for: Methodological Insight Into Mosquito Microbiome Studies
Source: Front Cell Infect Microbiol. 2020 Mar 17;10:86. doi: 10.3389/fcimb.2020.00086 (PMC7089923; doi:10.3389/fcimb.2020.00086)
Supplement: Supplementary file 5 [file Image_3.pdf]

Supplementary Material

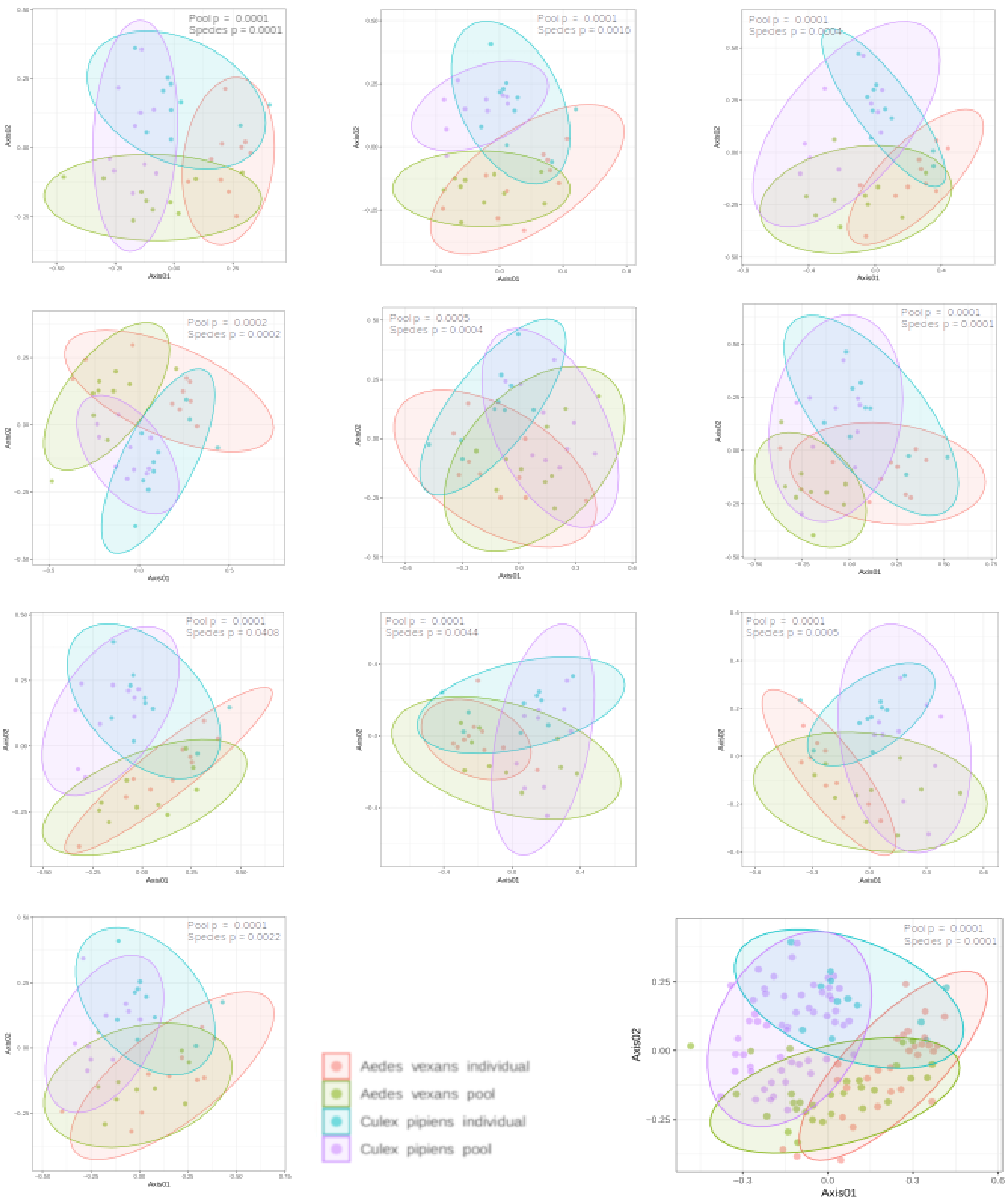

Supplementary Figure 3. NMDS showing the beta-diversity of *Aedes vexans* and *Culex pipiens* microbiomes both from individuals and pools, using 10 randomly sub-sampled data sets (n = 10 for each group). Confidence ellipses are shown for each group. The results for the full data set presented in the main text are also shown at the bottom-right of the figure for easier comparison.
