# Supplementary material for: Methodological Insight Into Mosquito Microbiome Studies
Source: Front Cell Infect Microbiol. 2020 Mar 17;10:86. doi: 10.3389/fcimb.2020.00086 (PMC7089923; doi:10.3389/fcimb.2020.00086)
Supplement: Supplementary file 6 [file Image_4.pdf]

Supplementary Material

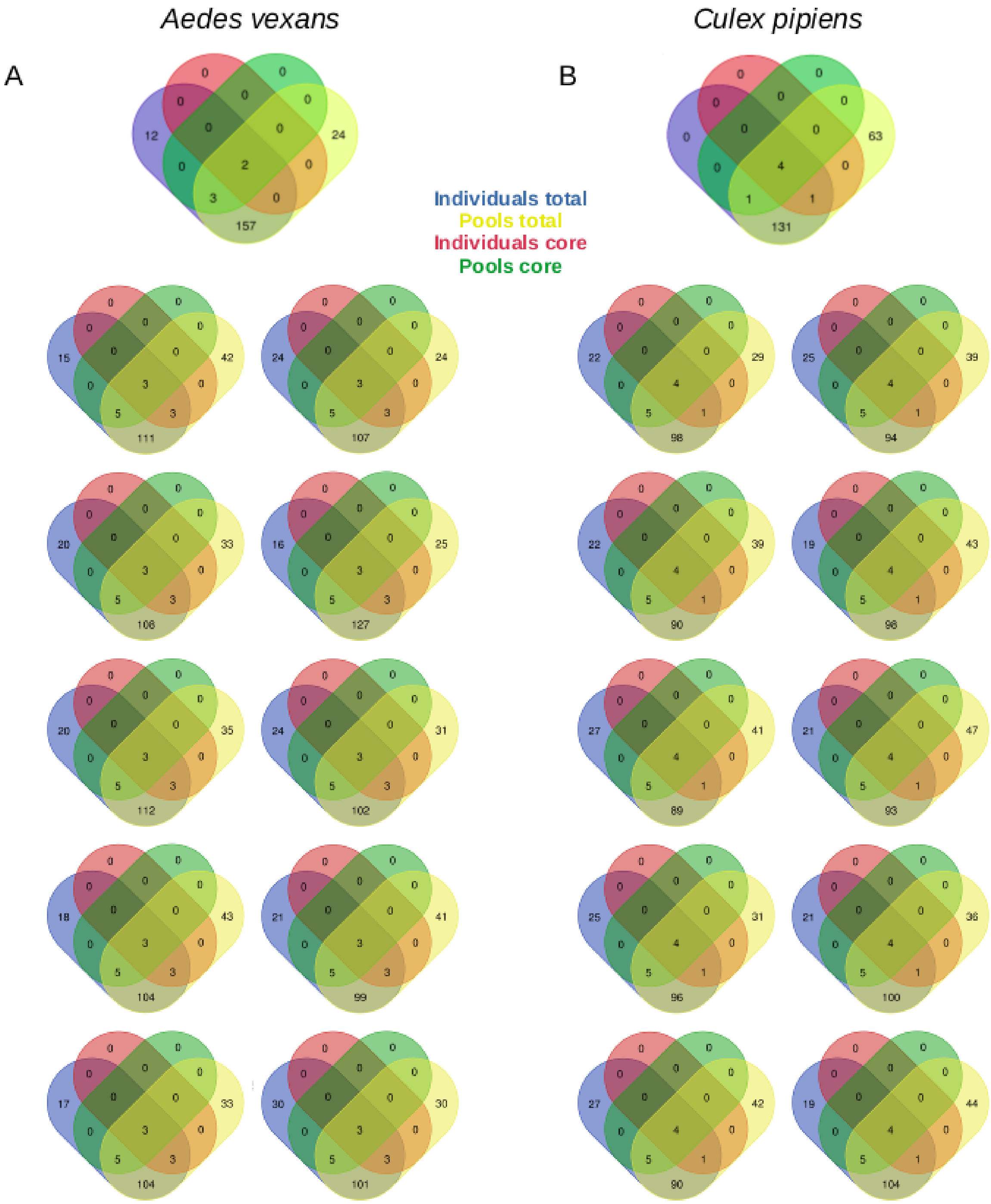

Supplementary Figure 4. Venn diagrams comparing the number of OTUs present in individual vs. pooled samples, both in total and in the core microbiome for A) *Aedes vexans* and B) *Culex pipiens*, using 10 randomly sub-sampled data sets (n = 10 for each group). The results for the full data set presented in the main text are also shown at the top of the figure for easier comparison.
